# Using the Electronic Medical Record to Identify Community-Acquired Pneumonia: Toward a Replicable Automated Strategy

**DOI:** 10.1371/journal.pone.0070944

**Published:** 2013-08-13

**Authors:** Sylvain DeLisle, Bernard Kim, Janaki Deepak, Tariq Siddiqui, Adi Gundlapalli, Matthew Samore, Leonard D'Avolio

**Affiliations:** 1 Department of Medicine, Veterans Affairs Maryland Health Care System, Baltimore, Maryland, United States of America; 2 Department of Medicine, University of Maryland, Baltimore, Maryland, United States of America; 3 Department of Medicine, Veterans Affairs Salt Lake City Health Care System, Salt Lake City, Utah, United States of America; 4 Department of Medicine, University of Utah, Salt Lake City, Utah, United States of America; 5 Massachusetts Veterans Epidemiology Research and Information Center, Veterans Affairs Boston Health Care System, Jamaica Plain, Massachusetts, United States of America; 6 Department of Medicine, Brigham and Women's Hospital, Harvard Medical School, Boston, Massachusetts, United States of America; University of Liverpool, United Kingdom

## Abstract

**Background:**

Timely information about disease severity can be central to the detection and management of outbreaks of acute respiratory infections (ARI), including influenza. We asked if two resources: 1) free text, and 2) structured data from an electronic medical record (EMR) could complement each other to identify patients with pneumonia, an ARI severity landmark.

**Methods:**

A manual EMR review of 2747 outpatient ARI visits with associated chest imaging identified x-ray reports that could support the diagnosis of pneumonia (kappa score  = 0.88 (95% CI 0.82∶0.93)), along with attendant cases with Possible Pneumonia (adds either cough, sputum, fever/chills/night sweats, dyspnea or pleuritic chest pain) or with Pneumonia-in-Plan (adds pneumonia stated as a likely diagnosis by the provider). The x-ray reports served as a reference to develop a text classifier using machine-learning software that did not require custom coding. To identify pneumonia cases, the classifier was combined with EMR-based structured data and with text analyses aimed at ARI symptoms in clinical notes.

**Results:**

370 reference cases with Possible Pneumonia and 250 with Pneumonia-in-Plan were identified. The x-ray report text classifier increased the positive predictive value of otherwise identical EMR-based case-detection algorithms by 20–70%, while retaining sensitivities of 58–75%. These performance gains were independent of the case definitions and of whether patients were admitted to the hospital or sent home. Text analyses seeking ARI symptoms in clinical notes did not add further value.

**Conclusion:**

Specialized software development is not required for automated text analyses to help identify pneumonia patients. These results begin to map an efficient, replicable strategy through which EMR data can be used to stratify ARI severity.

## Introduction

Effective responses to epidemics of infectious diseases hinge not only on early outbreak detection, but also on an ongoing assessment of disease severity. Indeed, the proportion of infected patients who develop severe illness often governs public perception and is a key factor in deciding whether or not to trigger interventions that can cause harm and exact significant social and financial costs.

For surveillance systems aimed at epidemics of acute respiratory infections (ARI), the rationale for incorporating information about disease severity is particularly compelling: 1) doing so could help discover outbreaks that involve only a small number of very sick patients, such as what initially occurred with SARS [1] or what could be anticipated shortly after a criminal release of plague [2] or tularemia [3]; 2) such systems could help adjust ongoing responses to seasonal or pandemic influenza, where severity can vary by orders of magnitude between epidemics [4] or even between waves of the same epidemic [5], [6]. To be useful, information about ARI severity needs to be both timely and specific [7], [8]. Current methods of monitoring influenza-related hospitalizations or deaths fall short of meeting these requirements [9].

Electronic medical records (EMR) are fast becoming commonplace, and form a rich source of information that could be secondarily used for surveillance purposes. In the past, we initiated a project to unravel how EMR data could be combined to identify outpatients with ARI [10]. In this work, we sought to develop case-detection algorithms (CDA) aimed at pneumonia, a key landmark in the severity spectrum of ARI. In particular, we asked how information retrieved from the free-text of chest imaging reports and clinical notes could complement structured data to uncover pneumonia cases.

## Methods

### Ethics Statement

This study was approved by the Institutional Review Boards at the University of Maryland and the VA Maryland Health Care System. Research-related risks were limited to maintaining the confidentiality of data generated during routine patient care. A waiver of consent was granted because the research-related risks were minimal and did not adversely affect the rights and welfare of the participants, and because the work would not have otherwise been feasible, given the large number of participants.

### Participants

We applied a previously validated ARI case-detection algorithm (CDA) [10] to EMR-derived information related to outpatient visits at the Veterans Administration Maryland Health Care System, from January 1, 2004 through December 31, 2006. This ARI CDA was chosen as a screening tool because it identifies 99% of outpatients that satisfied a broad definition of ARI: positive respiratory virus culture/antigen OR any two of the following symptoms, of no more than 7 days duration: a) cough; b) fever or chills or night sweats; c) pleuritic chest pain; d) myalgia; e) sore throat; f) headache AND illness not attributable to a non-infectious etiology [10]. The ARI CDA flagged an outpatient visit if the provider assigned it an ARI-related International Disease Classification, 9^th^ Revision, Clinical Modification (ICD-9) diagnostic code OR issued a prescription for a cough remedy OR documented at least two symptoms from the above ARI case definition in his/her clinical note, as retrieved by computerized text analysis [10]. Visits flagged by the ARI CDA were included if chest imaging was obtained within 24 hours of clinic registration time. Participants were sampled only once, at their first eligible visit during the study period.

The methods to validate the performance of selected pneumonia CDA on a separate population are described in the next section.

### Description of Procedures

#### Reference chest imaging report review

A pulmonary disease physician read all eligible chest imaging reports (n = 2,861 in 2747 unique patients). Reports were labeled “Negative” if they did not support the diagnosis of pneumonia. This category included all images within normal limits or showing no evidence of active pulmonary disease. Reports with comments on shrapnel or bullet fragments, pleural plaques or other abnormalities outside the lung parenchyma, calcified granulomas, old nodules, scars or chronic emphysematous changes were put in this category. Reports were labeled “Non-Negative” if they could possibly support the diagnosis of pneumonia. These reports described a wide range of abnormalities, from ill-defined densities where the diagnosis of pneumonia could not be excluded, to frank infiltrates characteristic of pneumonia. All “Non-Negative” reports and a 10% sample of the “Negative” reports were blindly reviewed by a second pulmonary physician (n = 537). Kappa score between the two independent reviewers was 0.88 (95% CI 0.82:0.93). “Non-negative” reports containing wording typically used to describe abnormalities indicative of pneumonia were also flagged and used as an alternative training set in the development of the automated imaging report classifier (see below).

#### Reference clinical record review

Reference cases with pneumonia were identified by manually reviewing all EMR entries made during the calendar day of index visits that corresponded to the reference, manually reviewed, “Non-Negative” chest imaging reports outlined above. Symptoms and diagnostic impressions were abstracted by a pulmonary physician, entered into a data collection instrument (MS Access, Microsoft Corp., Redmond WA) and recombined into two case definitions: 1) “Possible Pneumonia”: non-negative chest imaging report AND at least one of the following symptoms, new or changed within the last 7 days: a) cough; b) sputum; c) fever or chills or night sweats; d) dyspnea; e) pleuritic chest pain AND illness not clearly attributable to a non-infectious etiology; 2) “Pneumonia-in-Plan”: a non-negative chest imaging report AND pneumonia listed as one of the top two diagnostic possibilities in a physician's or nurse practitioner's note. Cases with Possible Pneumonia or Pneumonia-in-Plan were labeled “Admitted” if they gained admission to the hospital within 48 hours of index visit registration. Otherwise, they were labeled “Outpatient”.

#### Development of chest imaging report classifier

We used open-source automated software that couples a clinical NLP pipeline (Clinical Text Analysis and Knowledge Extraction System (cTAKES) [11]) with an implementation of a conditional random fields probabilistic classifier [12] to develop the text analyses that could separate non-negative from negative chest imaging reports (Automated Retrieval Console (ARC) software, v.2.0 [13], [14]). In a preliminary effort to improve the performance of the classifier, the reference imaging reports were presented for machine-learning as four alternative training sets where: a) the text of the reports was fed either whole or scrubbed from the characters preceding the string “Impression” when the latter was found; b) targeted reports were either all of the non-negative reports (n = 450) or only those that described abnormalities typical for a pneumonia (n = 316). Text classification models with the highest F-measure were retained for each training set. The four retained models were then separately combined with other EMR-derived data and performance of the resulting CDAs at identifying patients that fitted our case definition compared (see next paragraph). The text classification models trained with reports that contained typical pneumonia descriptions and whose text was restricted to the “Impression” field led to the best performing pneumonia CDAs, and were those used for this report.

#### Development of pneumonia case-detection algorithms

Data were extracted from the Veterans Integrated Service Technology Architecture (VistA) repository and transferred to a Structured Query Language (SQL) database (Mumps Data Extractor, Strategic Reporting Systems Inc., Peabody, MA). Subsequent data transformations and queries were implemented using SQL Server 2008 (v. 10.0, Microsoft Corp., Redmond, WA). Candidate components for CDAs included those previously found useful to identify patients with ARI: ARI-related ICD-9 codes (labeled as “ARI ICD-9 codes”), cough remedies [10], and clinical notes identified as positive for ARI symptoms by text analysis [10] (“Text of Clinical Notes”). We also considered the following CDA components, when related to the index outpatient visit: 1) a subset of the ARI ICD-9 codes whose narrative included the string “pneumonia” (“Pneumonia ICD-9 codes”: 480–483, 485–487); 2) a new prescription for antibiotics of a class of commonly used to treat pneumonia (cephalosporins, fluoroquinolones, macrolides, penicillins); 3) admission to the hospital, for any reason, within 48 hours of the index outpatient visit (“(Not) Admitted to Hospital”); 4) chest imaging performed (“Imaging Obtained”); 5) whether at least one chest imaging report related to the index visit was labeled “non-negative” by the automated text classifier described above (“Text of Imaging Reports”).

#### Performance measures

The performance of the pneumonia CDAs was summarized with standard test descriptors (sensitivity, specificity, positive predictive value (PPV), negative predictive value (NPV) and F-measure (2 * PPV * Sensitivity/(PPV + Sensitivity)). Denominators used to calculate these tests were either the whole study population (n = 2747), those patients who were hospitalized for any reason following their index visit (n = 602) or those who were not (n = 2145).

#### Validation of selected CDAs

The ARI CDA and imaging report classifier were applied to EMR-derived databases for a 5-year period anterior to the original study period i.e. 1/1/2007–12/31/2011. A random, 50% sample of the visits flagged by the [ARI CDA AND Text of Imaging Reports] query were manually reviewed. Cases identified served as the reference to validate the PPV of selected pneumonia CDAs.

## Results

### Study Population

The ARI CDA flagged 22,960 first visits from unique patients during the algorithm development phase of the study period. Of these, 2,747 were associated with at least one report for chest imaging performed within 24 hours of check-in time. The study population was 93% male, older (61±15 years old, mean ± standard deviation) and 52.6% African American (Table 1).

**Table 1:** Patient demographics.

	Study Population N (%)	Possible Pneumonia N (%)	Pneumonia-in-Plan N (%)	Admitted Pneumonia N (%)
Sample Size	2747	370	250	127
**Age Group (years)**				
<21	0	0	0	0
21-30	66 (2.4)	3 (0.8)	1 (0.4)	1 (0.8)
31–40	130 (4.7)	9 (2.4)	7 (2.8)	2 (1.6)
41–50	546 (19.9)	67 (18.1)	47 (18.8)	18 (14.2)
51–60	762 (27.7)	109 (29.5)	77 (30.8)	30 (23.6)
61–70	443 (16.1)	61 (16.5)	39 (15.6)	23 (18.1)
71–80	528 (19.2)	74 (20)	51 (20.4)	31 (24.4)
81–90	256 (9.3)	43 (11.6)	26 (10.4)	22 (17.3)
91–100	16 (0.6)	4 (1.1)	2 (0.8)	0
**Sex**				
Male	2560 (93.2)	355 (95.9)	243 (97.2)	125 (98.4)
Female	187 (6.8)	15 (4.1)	7 (2.8)	2 (1.6)
**Race**				
White	1013 (36.9)	132 (35.7)	93 (37.2)	49 (38.6)
African-American	1446 (52.6)	210 (56.8)	143 (57.2)	75 (59.1)
Hispanic-American	20 (0.7)	2 (0.5)	1 (0.4)	0
Other	268 (9.8)	26 (7.0)	13 (5.2)	3 (2.4)

The number of patients belonging to the various age, sex and self-reported race groups (rows) are given for the overall study population (column 2), patients with Possible Pneumonia (column 3), Pneumonia-in-Plan (column 4), and for patient admitted for pneumonia (column 5). Numbers in parenthesis indicate the percentage relative to the total for each demographic category.

### Reference Pneumonia Cases

A manual review of EMR entries on the day of the 2,747 index visits identified 380 cases that satisfied at least one pneumonia case definition, 370 with Possible Pneumonia and 250 with Pneumonia-in-Plan. Most patients with a Pneumonia-in-Plan also had Possible Pneumonia (240/250), including nearly all (124/127) patients admitted to the hospital. Patients who satisfied either case definitions were therefore merged into a common target group for the development of the “Admitted Pneumonia” CDAs. Ninety percent of all index visits occurred in urgent/same day care settings.

Patients with Possible Pneumonia and Pneumonia-in-Plan had similar demographics (Table 1) and symptoms and signs (Table 2), with the possible exception that the latter population had more febrile symptoms. Compared with their outpatient counterparts, Admitted Pneumonia patients were overrepresented in the older age groups (71–90 years old, Table 1) and appeared to have more dyspnea, fever-related symptoms, and clinical signs of lung consolidation (Table 2).

**Table 2:** Symptoms and Signs.

	Possible Pneumonia (All)	Pneumonia-in-Plan (All)	Possible Pneumonia (Outpatients)	Pneumonia-in-Plan (Outpatients)	Admitted Pneumonia
Sample Size	370	250	193	107	127
**Symptoms and Signs**					
Cough	287 (77.6)	196 (78.4)	162 (83.9)	88 (82.2)	94 (74)
Sputum production	196 (53)	140 (56)	111 (57.5)	68 (63.6)	62 (48.8)
Fever chills or night sweats	174 (47)	157 (62.8)	72 (37.3)	42 (39.3)	75 (59.1)
Dyspnea	156 (42.2)	103 (41.2)	67 (34.7)	27 (25.2)	71 (55.9)
Pleuritic chest pain	43 (11.6)	35 (14)	20 (10.4)	15 (14)	16 (12.6)
Rhinorrhea, sinus pain or tenderness	6 (1.6)	5 (2)	5 (2.6)	2 (1.9)	1 (0.8)
Sore, throat or tonsillar exudate	34 (9.2)	21 (8.4)	23 (11.9)	11 (10.3)	10 (7.9)
Myalgia	27 (7.3)	18 (7.2)	17 (8.8)	10 (9.3)	7 (5.5)
Headeache	26 (7)	15 (6.0)	13 (6.7)	4 (3.7)	8 (6.3)
Signs of lung consolidation	84 (22.7)	67 (26.8)	36 (18.7)	22 (20.6)	38 (29.9)

Absolute and relative (%, in parenthesis) number of pneumonia patients with symptoms that were part of our Possible Pneumonia definition (rows 3–7) or that commonly occur in ARIs (rows 8–11). Physical signs of lung consolidation (row 12) included documented rales, bronchial breathing, egophony and whispered pectoriloquy. Populations with Possible Pneumonia (column 2) and Pneumonia-in-Plan (column 3) are broken down into outpatients (columns 4 and 5) and admitted subpopulations (column 6).

### Pneumonia CDAs That Used Structured EMR Entries Only

The composition and performance of illustrative CDAs for cases with Possible Pneumonia or Pneumonia-in-Plan are shown for all locations of care in Table 3, and for those cases that remained outpatients or were admitted (Tables 4 and 5, respectively). Structured EMR information *ipso facto* included as components of the relevant CDAs included: 1) that chest imaging was obtained (“Imaging Obtained”, Tables 3–5); 2) whether or not a case was admitted to the hospital (“(Not) Admitted”, Tables 4–5).

**Table 3:** Performance of pneumonia CDAs: All outpatients.

CDA Number		1	2	3	4	5	6	7	8	9	10	11	12	13	14
Target Diagnosis		Possible Pneumonia							Pneumonia-in-Plan						
**CDA Components**	(Pneumonia ICD-9 Codes	•	•						•	•					
	(ARI ICD-9 Codes			•	•	•	•	•			•	•	•	•	•
	OR Text of Clinical Notes)						•	•						•	•
	AND Text of Clinical Notes)				•							•			
	AND Imaging Obtained	•	•	•	•	•	•	•	•	•	•	•	•	•	•
	AND Text of Imaging Reports		•		•	•		•		•		•	•		•
**Performance**	Sensitivity (%)	36.8	28.4	85.9	14.3	58.4	99.7	66.2	52	40.8	93.6	18.8	68.8	100	74.8
	Specificity (%)	95.4	99.7	29.8	99.7	98.5	2.2	98	95.4	99.6	29.8	99.5	96.8	2.3	95.7
	PPV (%)	55.3	93.8	16	89.8	86.1	13.7	83.3	52.8	91.1	12	79.6	68.5	9.3	63.6
	PPV Validation* (%)		81		83	79		78		73		65	61		57
	NPV (%)	91	90	93.2	88.2	93.8	98.1	95	95.2	94.4	98	92.4	97	100	97.4
	F-Measure	44.2	43.6	27.0	24.7	69.6	24.1	73.8	52.4	56.4	21.0	30.4	68.6	17.0	68.7

Composition and performance of CDAs at identifying outpatients with Possible Pneumonia (CDAs number 1–7) or Pneumonia-in-Plan (CDA number 8–14). A *black dot* indicates that a component (column 2) is included in the CDA (see text).

* CDA implemented on EMR data not used in CDA development i.e. from 1/2007–12/2011.

**Table 4:** CDA Performance: Outpatients who were not admitted to the hospital.

CDA Number		15	16	17	18	19	20	21	22	23	24	25	26	27	28
Target Diagnosis		Possible Pneumonia							Pneumonia-in-Plan						
CDA Components	(Pneumonia ICD-9 Codes	•	•						•	•					
	(ARI ICD-9 Codes			•	•	•	•	•			•	•	•	•	•
	OR Text of Clinical Notes)						•	•						•	•
	AND Text of Clinical Notes)				•							•			
	AND Imaging Obtained	•	•	•	•	•	•	•	•	•	•	•	•	•	•
	AND Text of Imaging Reports		•		•	•		•		•		•	•		•
	AND Not Admitted	•	•	•	•	•	•	•	•	•	•	•	•	•	•
Performance	Sensitivity (%)	28.5	23.3	90.2	14.5	61.1	99.5	65.8	46.7	39.3	97.2	21	72.9	100	75.7
	Specificity (%)	95	99.2	25	99.4	97.5	2.5	97.1	95	99.1	24.6	99.2	95.6	2.4	94.9
	PPV (%)	36.2	74	11	72	71.1	9.2	69	33	68.8	6.3	56.4	47	5.1	44
	PPV Validation (%)		69		75	71.4		74		60		50	49.5		49
	NPV (%)	93.1	93	96.2	92.2	96.2	98	97	97	96.8	99.4	95.9	98.5	100	98.6
	F-Measure	32	35.4	19	24.1	65.7	17	67.4	38.9	50	11.9	30.1	57.1	9.7	55.7

**Table 5:** CDA Performance: Outpatients subsequently admitted to the hospital.

CDA Number		29	30	31	32	33	34	35
Target Diagnosis		Admitted Pneumonia						
CDA Components	(Pneumonia ICD-9 Codes	•	•					
	(ARI ICD-9 Codes			•	•	•	•	•
	OR Text of Clinical Notes)						•	•
	AND Text of Clinical Notes)				•			
	AND Imaging Obtained	•	•	•	•	•	•	•
	AND Text of Imaging Reports		•		•	•		•
	AND Admitted	•	•	•	•	•	•	•
Performance	Sensitivity (%)	56	42	89	9.8	62.6	100	73.6
	Specificity (%)	92	97.5	48.5	97.8	94.5	0.8	91.5
	PPV (%)	54.3	74.5	23.5	45	67.1	15.2	61
	PPV Validation (%)		89		90	86		86
	NPV (%)	92.1	90.4	96.1	85.9	93.4	100	95.1
	F-Measure	55.1	53.5	37.2	16.2	64.7	26.4	66.7

An ICD-9 code set for pneumonia diagnoses (“Pneumonia ICD-9 Codes”, Tables 3–5) helped identify pneumonia with PPVs of 52.8–55.3% but had limited sensitivity (28.5–56%, CDAs 1, 8, 15, 22, and 29, Tables 3–5), even when providers had indicated that pneumonia was a likely diagnosis in their clinical notes i.e. in Pneumonia-in-Plan or Admitted Pneumonia cases (CDAs 8, 22 and 29, Tables 3–5). A broadly inclusive ARI ICD-9 code set (“ARI ICD-9 Codes”, Tables 3–5) increased detection sensitivity to 86–97%, but degraded PPV (6.3–23.5%) and overall performance, as reflected by lower F-measures (compare CDA 3 to 1, 10 to 8 in Table 3, 17 to 15, 24 to 22 in Table 4, and 31 to 29 in Table 5).

CDAs that did not include ICD-9 diagnostic codes were not among the most successful (data not shown). Prescriptions for medications aimed at ARI symptoms and various groupings of antibiotics that could be used to treat bacterial pneumonias did not add value (data not shown).

### Pneumonia CDAs That Combined Structured with Free-Text EMR Entries

We retrieved information from free-text EMR entries according to two different strategies. In the first strategy, text analysis routines were used to search for ARI symptoms in the providers' clinical notes (“Text of Clinical Notes”, Tables 3–5). Coupling positive results of Text of Clinical Notes analyses to ARI ICD-9 codes using an OR logical operand increased detection sensitivity over otherwise comparable CDAs. However, specificity and PPV decreased and overall performance either did not improve or worsened (compare CDA 6 to 3 and 13 to 10, Table 3; CDA 20 to 17 and 27 to 24, Table 4; CDA 34 to 31 and 35 to 33, Table 5). Coupling the Text of Clinical Notes analysis to ARI ICD-9 codes using an AND logical operand further increased PPV, but severely reduced sensitivities and overall performance (CDA 4, 11, 18, 25 and 32, Tables 3–5).

In the second strategy, text analysis was used to flag chest imaging reports that could support the diagnosis of pneumonia (“AND Text of Imaging Reports” component, Tables 3–5). Adding this component increased the PPV of otherwise identical CDAs by 23–70 absolute percentage points (compare CDA 2 to 1, 5 to 3, 7 to 6 and so on, Tables 3–5). Despite attendant losses in sensitivity, results from the “Text of Imaging Reports” classifier increased the F-measure of all CDAs that included the broad ARI ICD-9 code set. With the possible exception is CDA 7, whose F-measure was the highest achieved in this study, the OR Text of Clinical Notes component did not add further value to CDAs that already included analyses of the chest imaging reports (compare CDA 7 to 5 and 14 to 12, Table 3; CDA 21 to 19 and 28 to 26, Table 4; CDA 35 to 33, Table 5).

### Performance Validation

The [ARI CDA AND “Text of Imaging Report”] algorithm flagged 553 visits over the 1/2007–12/2011 validation time period. Of the 276 visits manually reviewed, there were 212 cases with Possible Pneumonia, 154 with Pneumonia-in-Plan and 147 with both diagnoses. These reference cases were used to measure the PPV of CDAs that included the “Text of Imaging Report” CDA component (“PPV Validation” performance measure, Tables 3–5). For the whole population, PPVs decreased 5.3–18% absolute percentages points compared to values obtained in the development phase of the study (compare PPV vs. PPV Validation performance measures for CDAs 2, 4, 5, 7, Table 3). For the best performing CDAs, PPVs remained within 5.3–7.5% of our original findings, independent of case definition and admission disposition (CDAs 5, 7, 12, 14, 19, 21, 26, 28, 33, 35, Tables 3–5). The added imprecision occurred mostly in the outpatient arena (CDA 19, 21, 26, 28, Table 4) and was in large part due to flagging of follow-up rather than initial pneumonia visits (data not shown). PPVs actually increased for patients admitted to the hospital (CDA 33, 35, Table 5).

## Discussion

Automated text analyses of chest imaging reports improved the performance of EMR-based CDAs that included structured data elements and free-text search for ARI symptoms. This contribution persisted across pneumonia case definitions, applied to outpatients and hospitalized patients alike, and helped CDAs reach precisions of 64–86% while maintaining sensitivities of 58–75%. These data support our working hypothesis that selected free text analyses can supplement structured EMR data to assess the severity of ARI outbreaks.

This work benefits from prior efforts to combine EMR data to identify patients with ARI. The ARI CDA used as an initial screen for the current study had been developed and validated against a population-based sample of over 15,000 EMR records, where it recognized 99% of cases that satisfied a broad definition of ARI [10]. This screening algorithm forms a practical starting point for an EMR data flow intent on monitoring the incidence and severity of ARIs, and is likely to have flagged most symptomatic pneumonia patients.

Pneumonia is seldom a definitive diagnosis, even when histological information is available [15]. Absent a standard, we sought clinically acceptable case definitions that could be reliably abstracted from clinical records. As is both customary and recommended by treatment guidelines [16]–[19], our case definitions required supportive chest imaging. To this common imaging requirement, the Possible-Pneumonia definition added clinical symptoms whereas Pneumonia-in-Plan relied solely on the provider's final diagnostic assessment. Despite these differences, more than 95% of patients with Pneumonia-in-Plan also satisfied the more permissive Possible Pneumonia definition in both our development and validation reference populations, indicating that the two definitions addressed related clinical conditions. Given that independent EMR abstractors could identify respiratory symptoms [10], pneumonia diagnostic impressions and supportive chest imaging with a high degree of agreement, our data suggest that the Possible Pneumonia and the Pneumonia-in-Plan case definitions can serve as useful tools to reproducibly retrieve pneumonia-related information from an EMR.

Prior attempts to automatically identify pneumonia patients through medical records have concentrated on diagnostic codes assigned after hospital discharge. Discharge codes have been found to be good markers for hospitalized pneumonia patients, whether benchmarked against retrospective record reviews [20]–[22] or prospective data acquired for clinical trials [23]–[26]. Discharge codes, however, are of limited value in epidemic surveillance because they are untimely and do not distinguish between community- and hospital-acquired pneumonia [22]. In this study, we evaluated diagnostic codes assigned by providers at the conclusion of outpatient visits, as is practiced at the Veterans Administration health system. We found these codes to represent a key component of pneumonia detection, even if they proved less accurate at finding pneumonia patients who were sent home rather than hospitalized [27]. While the utility of diagnostic codes vary when they are assigned by third parties or have reimbursement repercussions, our results nevertheless provide an impetus for diagnostic codes to be made available as soon as possible following outpatient services, so that they can be used for surveillance, decision support and quality control.

The chest imaging report has long been recognized as a fruitful context in which to mine for evidence of pneumonia. Over the last 20 years, various combinations of approaches, including natural language processing [28]–[31], expert rules [32], [33], Bayesian [32], [34] or neural networks [35] and machine-learning [33], have held their own compared to physicians for their ability to find pneumonia-related concepts in report narratives. Imaging report analyses have been compared to discharge diagnostic codes [36], [37], but have seldom been evaluated for their added value against a broader reference standard for clinical pneumonia [38]–[40]. To our knowledge, only one previous publication used imaging report analyses to detect outpatients with community-acquired pneumonia [40]. Besides bolstering the evidence for the utility of these text analyses, our data illustrate the importance of targeting them properly: in the course of this study, classifying 26,581 imaging reports did more to improve detection performance than extracting ARI symptoms from almost 14 million clinical notes. Although an assessment of the significance of the performance gained through imaging report text analysis must await purpose-specific evaluations, our data nevertheless support the notion that a generalized machine learning approach can perform well across information retrieval tasks [13], [14]. Also significant, in our view, is the ease with which we could develop the classifier. Clinical users focused on the document-level classification needed to create the reference training set. Once the latter was fed to the ARC software, model development required little further user interaction, and there was no need for custom programming. Such an efficient workflow makes it possible to quickly rebuild the classifier elsewhere, should it proves less robust than our validation exercise suggests.

Our study is subject to limitations beyond those already mentioned. First, we did not evaluate CDA components that have been associated with pneumonia in the past such as abnormalities in vital signs [41], white blood cell count [42] or oxygenation [41], and microbiological results. While these data elements could be missing in some patients [43], they could provide an opportunity to further improve detection performance. Second, our work was performed in a health system whose population and health care practices may not be generalizable. Even if diffusion of our approaches was initially restricted to VA institutions, at least some automated pneumonia surveillance could nevertheless be deployed across all 50 states. Third, sampling was not random but instead based on a screening algorithm. While this algorithm has been validated using a random, population-based sample, our study sample remains subject to verification bias [44] such as the systematic exclusion of pneumonia patients for whom chest imaging was not obtained [45]. Fourth, the retrospective nature of the record review coupled with shortcomings of clinical acumen and chest imaging [46] imply that we may have missed pneumonia patients whose symptoms, signs or imaging abnormalities were absent [46], [47], missed, atypical, inadequately documented or miscoded [23]. Despite these potential failings, our results do reflect information committed to a real-world EMR, and thus represent a realistic environment in which to compare the relative performance of alternative detection approaches.

In summary, our results indicate that an EMR-based approach that couples queries of structured data with text analysis of imaging reports can be used to assess disease severity in outpatients with ARI. By identifying high-performing yet parsimonious CDAs that could be replicated without creating customized software, our results begin to map an efficient strategy by which pneumonia surveillance could be more widely implemented.
